# An Investigation into the Critical Factors Influencing the Spread of *Campylobacter* during Chicken Handling in Commercial Kitchens in China

**DOI:** 10.3390/microorganisms9061164

**Published:** 2021-05-28

**Authors:** Honggang Lai, Yuanyue Tang, Fangzhe Ren, Zeng Li, Fengming Li, Chaoyue Cui, Xinan Jiao, Jinlin Huang

**Affiliations:** 1 Jiangsu Key Lab of Zoonosis/Jiangsu Co-Innovation Center for Prevention and Control of Important Animal Infectious Diseases and Zoonoses, Yangzhou University, Yangzhou 225009, China; lhghstc@163.com (H.L.); tangyy@yzu.edu.cn (Y.T.); fzren@yzu.edu.cn (F.R.); lfm1009813208@163.com (F.L.); 18036195236@163.com (C.C.); jiao@yzu.edu.cn (X.J.); 2 College of Food Science and Engineering, Yangzhou University, Yangzhou, 225001, China; 3 Joint International Research Laboratory of Agriculture and Agri-Product Safety, Yangzhou University, Yangzhou 225009, China; 4 Key Laboratory of Prevention and Control of Biological Hazard Factors (Animal Origin) for Agri-food Safety and Quality, Ministry of Agriculture of China, Yangzhou University, Yangzhou 225009, China; 5 Jiangsu College of Tourism, Yangzhou 225000, China; jsyzlz123@163.com

**Keywords:** *Campylobacter*, chicken handling, sanitary conditions, MLST, biofilm

## Abstract

Campylobacteriosis is the most common cause of bacterial gastroenteritis worldwide. Consumption of chicken meat is considered the main route for human infection with *Campylobacter*. This study aimed to determine the critical factors for *Campylobacter* cross-contamination in Chinese commercial kitchens during chicken handling. Five commercial kitchens were visited to detect *Campylobacter* occurrence from 2019 to 2020. Chicken samples (n = 363) and cotton balls from the kitchen surfaces (n = 479) were collected, and total bacterial counts and *Campylobacter* spp. were detected. Genotypic characterization of 57 *Campylobacter jejuni* isolates was performed by multilocus sequence typing (MLST). In total, 77.41% of chicken carcass samples and 37.37% of kitchen surfaces showed *Campylobacter* spp. contamination. Before chicken preparation, *Campylobacter* spp. were already present in the kitchen environment; however, chicken handling significantly increased *Campylobacter* spp. prevalence (*p <* 0.05). After cleaning, boards, hands, and knives still showed high bacterial loads including *Campylobacter* spp., which related to poor sanitary conditions and ineffective handling practices. Poor sanitation conditions on kitchen surfaces offer greater opportunities for *Campylobacter* transmission. Molecular typing by MLST revealed that *Campylobacter* cross-contamination occurred during chicken preparation. The most prevalent sequence types, ST693 and ST45, showed strong biofilm formation ability. Consequently, sanitary condition of surfaces and biofilm formation ability of isolates were the critical points contributing to spread of *Campylobacter* in kitchen environment. These results provide insight into potential targeted control strategies along the farm-to-plate chain and highlight the necessity for improvements in sanitary conditions. The implementation of more effective cleaning measures should be considered to decrease the campylobacteriosis risk.

## 1. Introduction

*Campylobacter* is an important foodborne pathogen causing bacterial gastroenteritis worldwide [1]. Approximately 1 million individuals are infected by *Campylobacter* each year with an overall incidence of 16.5 cases per 10,000 in the United States [2,3], 64.8 cases per 10,000 in Europe [1], and 37.3–161.4 cases per 10,000 in China [4]. It was estimated that the annual economic cost of campylobacteriosis is approximately USD 6879 million in the United States [5] and EUR 2.4 billion in Europe [6]. Poultry meat is the main host of *Campylobacter* transmission, with 20%–30% of human *Campylobacter* infections originating from the processing, preparation, or consumption of chicken [7]. During food preparation in domestic kitchens, there are two main transmission routes resulting in human ingestion: (i) the cross-contamination route, where *Campylobacter* avoid heating by transmission through taps, hands, and raw vegetables, and (ii) the heating route, where pathogens remain on the chicken meat but are only partly inactivated by heating [8].

In China, poultry meat is the second most consumed type of meat (>20%) [9]. Chicken handling in commercial kitchens is complicated, and ineffective procedures and precautions can lead to consumer exposure to *Campylobacter* infection. In the kitchen environment, surfaces for poultry preparation include cutting boards, knives, and the handler’s hands [10]. A previous study identified the ability of foodborne pathogens to transmit from contaminated foods, such as chicken meat, to hands and direct contact surfaces in the domestic kitchen environment [11]. Although the cross-contamination and transfer rates for *Campylobacter* spp. have been well studied in domestic kitchens [12,13,14,15], the cross-contamination process is complicated and might be affected by many factors related to the characteristics of the bacteria, the contact surfaces, and processes employed [8]. Furthermore, much remains unknown about the cross-contamination risks during chicken preparation in commercial kitchens in China. Therefore, it is crucial to identify the critical point for the occurrence of *Campylobacter* in kitchen environments.

*Campylobacter* grows optimally at 37–42 °C under microaerobic conditions and cannot tolerant dryness or oxygen. Despite its requirements for fastidious growth conditions, it can also survive under adverse conditions, indicating that *Campylobacter* spp. may develop protective mechanisms. Adhesion to abiotic surfaces and biofilm formation is one of the main strategies for *Campylobacter* survival in the environment [16,17,18]. It has been reported that surface attachment and biofilm formation contribute to the persistence of *Campylobacter jejuni* on food and in the environment for relatively long periods [19].

This study aimed to determine the critical factors that contribute to the transmission of *Campylobacter* in commercial kitchen environments during chicken handling. This was achieved by quantifying the contamination loads by determining the total bacterial counts and the presence of *Campylobacter* spp. on raw chicken carcasses and kitchen surfaces and investigating the transmission potential of *Campylobacter* by multilocus sequence typing (MLST) and a biofilm formation test.

## 2. Materials and Methods

### 2.1. Sample Selection and Experimental Design

#### 2.1.1. Sample Procedure

Five commercial kitchens were selected for sampling from March 2019 to January 2020 in Yangzhou, Jiangsu province, China. A total of 33 visits for sample collection were performed in commercial kitchens, as shown in Table S1. During the sampling in commercial kitchens, chefs were asked to handle chicken carcasses according to their normal daily preparation routines in the raw meat preparation area. Samples were taken at all sampling sites before and after handling the chicken. The sampling sites included countertops, knives, cutting boards, hands, floors, sinks, and containers. Chicken carcasses were sampled at the stage of defeathering, evisceration, and washing at a sink in washing area. While handling chicken, chefs usually grasped chicken carcasses with their left hands. This hand was immediately wiped with two sterilized cotton balls when the chicken handling was finished. Following cleaning procedures, chefs washed food contact surfaces (such as knives, hands, and boards) with running tap water before we conducted the sampling.

#### 2.1.2. Sample Collection

The sampling method for chicken carcasses was carried out according to the HACCP regulation of the food safety inspection agency of the U.S. Department of Agriculture [20]. For chicken carcasses, two sterilized cotton balls soaked in physiological saline solution were used to wipe 250 cm^2^ of the exterior surface and 250 cm^2^ of the interior surface. For kitchen surfaces, two sterilized cotton balls soaked in physiological saline solution were used to wipe a surface area of approximately 100 cm^2^ including knives, boards, containers, countertops, floors, and sinks.

#### 2.1.3. Campylobacter Examination Method and Total Bacterial Count Analysis

The enumeration and isolation of *Campylobacter* spp. were carried out using the plating method [21]. The original solution was diluted 10× in PBS solution, and 100 μL of each dilution was spread onto *Campylobacter* selective agar base (modified CCDA, Preston; Oxoid, UK) plates containing selective antibiotics [22] and incubated under microaerophilic conditions (5% O_2_, 10% CO_2_, and 85% N_2_) at 42 °C for 48 h. Each dilution was plated twice. Clothes were homogenized with 225 ml of Buffered Peptone Water. Then, a 1 mL aliquot of the homogenate was inoculated into 9 mL of Bolton broth along with 5% defibrinated sheep blood and was incubated at 42 °C under microaerophilic conditions for 48 h. All colonies displaying *Campylobacter* colony morphology were counted within the countable dilution range (15–300 colony-forming units, CFU, per plate) for the presumptive quantification of initial *Campylobacter* contamination for each sample [23]. At least five *Campylobacter* colonies were selected for further identification. Primers used in multiplex PCR are listed in Table S2 [24]. PCR was performed according to a previous publication [25] to amplify the *16S rRNA* gene for all species, the *mapA* gene for *Campylobacter jejuni*, and the *ceuE* gene for *Campylobacter coli*. The quantification of *Campylobacter* by colony counting was conducted according to a previous report [26]. Plate count agar (PCA) was used to count the total microorganisms [27]. Each diluent (100 μL) was coated onto the appropriate plate, and the plates were incubated at 37 °C (±1 °C) for 24 h.

#### 2.1.4. Multilocus Sequence Typing (MLST)

Kitchen A, as a representative kitchen, was selected to investigate the transmission of *C. jejuni* during chicken carcass handling. In total, 57 *C. jejuni* isolates were identified from chicken and food contact surfaces in kitchen A. Multilocus sequence typing (MLST) was performed on all isolates from kitchen A to investigate the contamination route of *Campylobacter*. The MLST analysis was conducted according to a previous report [28]. Briefly, PCR amplification of seven housekeeping genes *aspA*, *glnA*, *gltA*, *glyA*, *pgm*, *tkt*, and *uncA* was conducted according to the guidelines provided on the *Campylobacter* MLST website (https://pubmlst.org/campylobacter/primers, accessed on 02 July 2020). PCR products were sequenced with an ABI 3730 automated DNA sequencer in both orientations. Sequence types (STs) and allele numbers were assigned using the *Campylobacter* MLST website (developed by Keith Jolley, University of Oxford; http://pubmlst.org/campylobacter, accessed on 11 August 2020).

#### 2.1.5. Biofilm Formation

Biofilm formation of *C. jejuni* strains was measured on 96-well polystyrene microtiter plates. Each *C. jejuni* strain was cultured in Brucella broth (Oxoid) to an absorbance value (A570) of 0.05 (∼10^7^ CFU/ml), and then 200 μL of the culture was transferred to a polystyrene microplate. After 72 h incubation, surfaces were washed with 200 µL of sterile water three times and dried at 60 °C for 30 min. Next, 100 μL of 0.1% (*wt/vol*) crystal violet solution was added and then incubated at 37 °C for 30 min. The unbound dye was removed by rinsing in sterile water and drying at 60 °C for 30 min. The bound crystal violet was dissolved by adding 20% acetone/80% ethanol, followed by incubation at room temperature for 15 min [29]. Then, the dissolved solution was measured at a wavelength of 570 nm with an Epoch spectrophotometer (BioTek, Winooski, VT, USA) using the Gen5 2.00 software. All experiments were performed using three technical replicates. Three wells were subject to the same treatment but without bacterial inoculation as the negative controls. The cutoff OD value (ODc) was defined as two times the negative control value, as previously reported [30]. Based on the OD values, strains were classified into the following three categories: non-biofilm producer (OD ≤ ODc), weak biofilm producer (ODc < OD ≤ 2 × ODc), and strong biofilm producer (2 × ODc < OD).

#### 2.1.6. Statistical Analysis

Data were analyzed using IBM SPSS v.21. Variances in the positive rate of detection of *Campylobacter* were compared between chicken carcasses and sample sites using the chi-square test. For all statistical comparisons, a level of significance of 0.05 was applied.

## 3. Results

### 3.1. Occurrence of Campylobacter spp. in Commercial Kitchens

The prevalence of *Campylobacter* in chicken carcasses and on sampling surfaces at each kitchen is presented in Table 1. Of the chicken carcasses tested, 281 of 363 (77.41%) were positive for *Campylobacter*. A total of 479 samples were collected from sample sites, including the handler’s hands, knives, boards, containers, countertops, floors, and sinks, and of these, 179 samples tested positive for *Campylobacter* (37.37%).

### 3.2. Occurrence of Campylobacter spp. and the Sanitary Conditions in Commercial Kitchens

In total, 58.57% of chicken carcasses were *Campylobacter* positive at the defeathering stage (Table 2), while the prevalence of *Campylobacter* increased to 87.84% at the evisceration stage (*p <* 0.001). At the washing stage, the prevalence of *Campylobacter* decreased to 79.91% (*p >* 0.05). Quantitative data indicated that the total bacterial count on the chicken carcasses during defeathering was 6.91 ± 1.20 log CFU/100 cm^2^, which was much higher than during evisceration and washing (*p* < 0.05). Furthermore, the *Campylobacter* count on chicken at the defeathering stage was the highest, reaching 2.67 ± 0.90 log CFU/100 cm^2^.

We collected 186 wiped samples from seven sample sites before chicken handling, and 14.52% of these samples were *Campylobacter* positive (Table 2). The prevalence of *Campylobacter* among the seven sampling sites ranged from 3.85% to 26.92%. After chicken handling, a total of 228 wiped samples were collected (Table 2) and the prevalence of *Campylobacter* among these samples significantly increased to 54.39% (*p* < 0.001). The prevalence of *Campylobacter* among the seven sampling sites ranged from 14.81% to 82.86%. For all food contact surface (FCS) sampling locations, including containers, hands, knives, and boards, the prevalence rates of *Campylobacter* were 82.86%, 68.57%, 57.14%, and 77.14%, respectively, and were significantly higher than for non-food contact surface (NFCS) sampling locations, including floors, countertops, and sinks (*p* < 0.05).

Following cleaning procedures, a total of 65 samples were obtained from knives, boards, and hands, with a positive rate of 43.08%. The rates of prevalence of *Campylobacter* on these surfaces significantly decreased to 45.45%, 52.17%, and 30%, respectively (Table 2).

Food contact surfaces, both before and after chicken handling, were the most contaminated areas, and in particular, chicken at the defeathering stage was highly contaminated (Table 3). Total bacterial counts were highest on boards, before and after chicken preparation, as well as after cleaning, with loads of up to 7.03 ± 1.10, 7.53 ± 0.74, and 7.04 ± 0.83 CFU/100 cm^2^, respectively, which were markedly higher than other contact surfaces. After chicken handling, the total bacterial counts for sample sites, especially hands, boards, and containers, significantly increased (*p* < 0.05), with the loads of *Campylobacter* being the highest on sinks and boards, at 3.04 ± 0.56 and 2.86 ± 0.18 log CFU/100 cm^2^, respectively. Following cleaning procedures, the *Campylobacter* load and the total bacterial counts on boards, hands, and knives did not significantly decrease (*p* > 0.05).

### 3.3. MLST Analysis of C. jejuni Isolates and the Analysis of Cross Contamination

**Figure 1:**
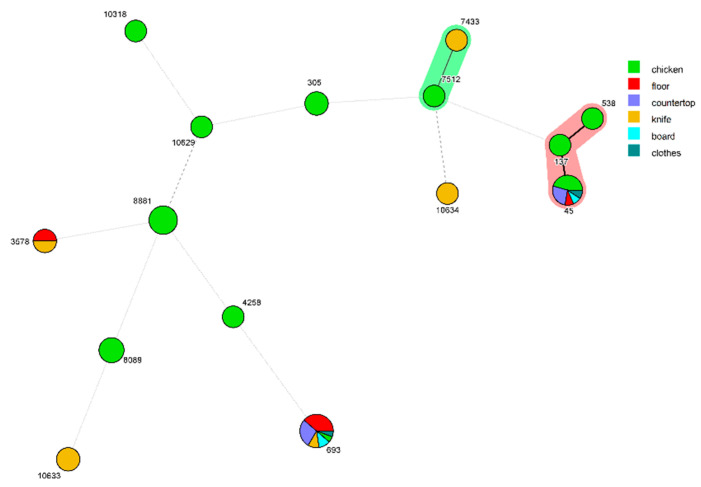
Minimum spanning trees based on MLST analyses of 57 *C. jejuni* isolates from chicken and sample sites from a commercial kitchen. Isolates are represented by circles connected by branches proportional to the allelic distance. The number represents the sequence type. Green represents isolates from chicken, red represents isolates from the floor, purple represents isolates from countertops, light brown represents isolates from knives, cyan represents isolates from boards, and dark green represents isolates from clothes.

Fifty-seven *C. jejuni* isolates were selected for MLST (Table S3). MLST characterization of these isolates yielded 16 known sequence types (STs). The most prevalent STs were ST693 (31.58%) and ST45 (19.3%). The MLST distribution of isolates sampled before and after chicken handling was concentrated on one or two STs, indicating that ST693 and ST45 were the most prevalent STs. Two isolates separately belonging to ST693 and ST3578 were isolated from the cleaning procedure. ST693 was isolated from chicken, knives, boards, floors, countertops, and clothes, whereas ST45 was isolated from chicken, boards, floors, countertops, and clothes (Figure 1). Interestingly, these two STs were observed in chicken samples at the washing stage.

### 3.4. Biofilm Assessment of Identified C. jejuni Isolates

**Figure 2:**
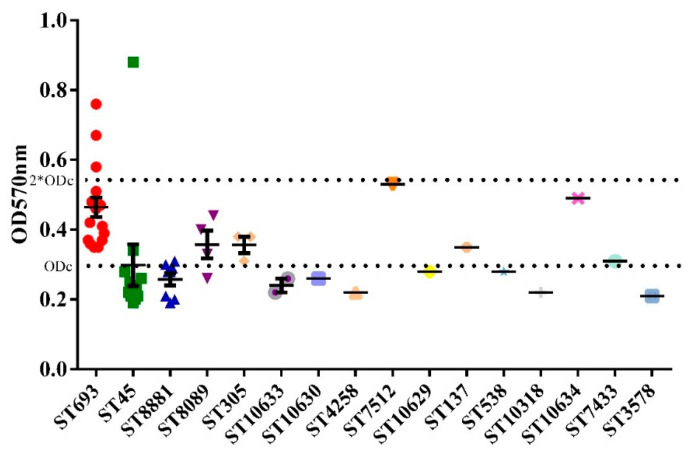
Biofilm-forming abilities of 57 *Campylobacter* isolates belonging to different sequence types. The bottom dotted line indicates the cutoff value (ODc = 0.2835), and the top dotted line indicates the two-fold cutoff value (ODc = 0.567). Based on the OD values, the strains were classified into three categories: non-biofilm producer (OD ≤ ODc), weak biofilm producer (ODc <OD ≤ 2 × ODc), and strong biofilm producer (2 × ODc < OD).

The degree of biofilm formation associated with the different MLST profiles is shown in Figure 2. Three isolates belonging to ST693 and one isolate belonging to ST45 showed strong biofilm formation ability. Although all of the strong biofilm producers belonged to ST693 or ST45 strains, these two STs exhibited different biofilm formation abilities, which suggested that the ability to form biofilm varied among the dominant genotypes of *Campylobacter*. Strains of ST7512, ST10634, ST7433, and ST3578, which were also isolated from the environment, showed weak biofilm formation, while other STs that showed no biofilm formation were not detected in the environment.

## 4. Discussion

This study focused on the prevalence of *Campylobacter* on chicken carcasses and sample sites in Chinese commercial kitchens, thereby providing data on the potential contamination of *Campylobacter* during chicken preparation. The results indicated that *Campylobacter* contamination is a potential public health concern in commercial kitchens. This study is the first to report the occurrence of *Campylobacter* during chicken handling in commercial kitchens in China.

Some previous studies have focused on *Campylobacter* contamination in domestic kitchens. The occurrence of *Campylobacter* on chicken in domestic kitchens was reported to be 77.8% (14 of 18) in Portugal [31], 44% (11 of 25) in Ireland [11], and 7%–35% in the UK [32]. In addition, a few studies have reported the occurrence of *Campylobacter* on chicken in commercial kitchens. *Campylobacter* was detected on the external surface of 88% (30 of 34) of fresh chickens at a commercial kitchen in the UK [33], and on only 9.7% of raw chickens in a Canadian food service establishment [34]. These results demonstrated that there was no difference in the *Campylobacter* detection rate between chicken carcasses at domestic and commercial kitchens, indicating that the chickens may be obtained from the same source and undergo similar processes during transportation and storage. In this study, a high number of samples were tested, and the rate of isolation of *Campylobacter* on chicken from five commercial kitchens ranged from 50% to 78.43%. One study simulated the evisceration of New York dressed poultry, considered an enteric ‘marker’ organism in a simulated kitchen environment, and showed that evisceration of chicken could cause greater contamination of the abdominal cavity and breast [35]. In our study, evisceration of chicken caused more *Campylobacter* cross-contamination of chicken samples in commercial kitchens, which indicated that the model of chicken processing in commercial kitchens should be changed to lower the occurrence of bacteria.

Medeiros and colleagues demonstrated the role of cross-contamination in *Campylobacter* propagation from raw poultry meat in a food service establishment, but *Campylobacter* was not detected from the surfaces in the kitchen environment [34]. To date, no study had investigated the contamination of *Campylobacter* in Chinese commercial kitchens. In this study, seven sample sites were selected, including knives, hands, boards, containers, floors, sinks, and countertops. Before chicken handling, the occurrence of *Campylobacter* on sample sites was 14.52% in the five commercial kitchens, indicating that *Campylobacter* was already present in the kitchen. After chicken handling, the prevalence of *Campylobacter* on the sample sites significantly increased to 54.39% (*p* < 0.05), which indicated that *Campylobacter* could transmit from chicken to kitchen surfaces. As expected, the food contact surfaces (knives, boards, hands, containers) were more contaminated than the non-food contact surfaces (countertops, floors, sinks). An earlier study indicated the presence of *Campylobacter* in wet cutting boards in Hong Kong markets [36]. Similarly, another previous study showed that kitchen utensils and boards were the main cross-contamination media [37]. The current study indicated that boards and containers were the most frequent sites contaminated by *Campylobacter* in commercial kitchens.

Dharod and colleagues concluded that after chicken handling, bacterial loads on countertops and boards were positively correlated to the loads on the handlers’ hands, but *Campylobacter* was not detected on any of the surfaces in their study [38]. Another study supposed that this failure to detect *Campylobacter* may be related to conventional cleaning of kitchen surfaces, proper storage of food, good hygiene, physical partitioning of raw and cooked foods, and the fragility of *Campylobacter* in dry conditions [34]. In the current study, after chicken handling, the counts of *Campylobacter* on surfaces (board, sink, knife, hand, and container) vary more greatly than other sites (knife, and hand), which indicated that the contamination of *Campylobacter* was heavy (Table 3). Boards were the sample site that showed the highest load of *Campylobacter*. Previous studies have already demonstrated that wooden cutting boards transfer a higher quantity of *C. jejuni* than plastic cutting boards, mostly because wooden boards are more porous than plastic boards, contributing to higher attachment of bacteria and therefore greater bacterial transfer [39,40]. It was also observed that hands, which were used frequently to handle meat and vegetables, showed high loads of total bacteria and *Campylobacter*. Interestingly, sinks in the vicinity of chicken evisceration acquired a large quantity of *Campylobacter*, despite the lack of direct contact between the sink surface and the chicken carcass. It is thought that sinks might be contaminated by water droplets or particles during hand washing [41]. Due to the lack of cooked food samples in this study, we did not obtain any evidence indicating that *Campylobacter* could transmit from raw chicken to cooked food by the cross-contamination route; this should be the focus of a future study.

It was noted in a previous study that surfaces that do not appear dirty can harbor a high bacterial load [31]. However, the total bacterial count has been regarded as a poor predictor of foodborne disease contamination in a kitchen environment [42]. In the current study, the CFU values of the total bacterial counts for all surfaces were higher than those required by the American Public Health Association [43]. In kitchen environments, the total bacterial counts for samples from FCS areas (hands, boards, and containers) were significantly higher than for NCFS areas (Table 3, *p* < 0.05). The fact that these surfaces were the most contaminated probably reflects the frequency of use (knives, containers, hands) or that the material was difficult to clean (boards). Following cleaning procedures, the hygiene condition of hands, knives, and boards was not obviously improved. In particular, boards that were directly in contact with chicken carcasses contained the highest bacterial loads after cleaning procedures, which may be explained by cross-contamination or ineffective hygiene practices. The poor hygiene status of most kitchen surfaces in this study can be attributed to *Campylobacter* cross-contamination between chicken and kitchen surfaces and the subsequent growth of microorganisms in biofilms [44]. Contamination with *Campylobacter* was highly consistent with the hygiene status of surfaces. Resident microorganisms have been reported to synergistically interact with one another, which strengthens their mode of survival [45]. Poor sanitary conditions on kitchen surfaces may be a critical factor allowing for the transmission of *Campylobacter*. Good hygiene practices in kitchen environments are therefore crucial to reduce the presence of *Campylobacter*. Therefore, some effective measures should be applied in the cleaning procedure, such as washing boards and knives with hot water or detergent and washing hands with hand sanitizer.

In a study on *Campylobacter* prevalence in Brazil, Gomes and coworkers found that ST353 was the most prevalent ST with seven strains isolated from humans and chicken meat, and ST8741 was the second most prevalent ST with three strains being isolated from humans and two strains being isolated from food [46]. In Sweden, ST45, ST48, and ST50 were dominant among chicken and human samples in 2000 and 2008 [47]. Zbrun and colleagues described a genotyping study of a collection of *C. jejuni* (n = 137) isolates from different broiler farms and slaughterhouses in Argentina and found that the most prevalent complex clones were 21 and 45 (both related to human campylobacteriosis worldwide) and 353 [48]. Our previous study found that the main ST of isolates from humans and food was ST353. In another study, the dominant ST in chicken and other food was ST354 [49]. However, in the current study, ST693 and ST45 were the most prevalent STs in the kitchen environment and among chicken samples at stage of washing, which indicated that the washing of chicken was critical to the spread of *Campylobacter* in kitchen environment. In a report of the genotypes of *C. jejuni* in Shenzhen, China, clonal complex ST45 was found in isolates from patients and poultry [50]. Another study also found the ST45 complex in raw or undercooked meat in Finland, and a variety of other STs were also found in chicken meat [51]. We could infer that ST45 is a likely cause of human infection through cross-contamination or eating uncooked meat in China. Although there have been few reports on ST693, interestingly, ST693 is more easily spread than ST45 in the kitchen environment and therefore might present a greater risk of human infection than ST45 via the cross-contamination route, which is worthy of further study.

By molecular characterization analysis and the use of qualitative and quantitative data collected during chicken handling, it was possible to establish cross-contamination from contaminated chicken carcasses to the kitchen environment. Interestingly, isolates collected from boards, knives, floors, countertops, and clothes all showed the same ST as the isolates from chicken samples in kitchen A (Figure 1, ST693 and ST45, respectively). Hence, it is possible to infer that the probable source of cross-contamination was the chicken carcass, which was consistent with data from a previous study [31]. A previous study also demonstrated the occurrence of cross-contamination when handling chicken in the home kitchen environment [11]. Furthermore, it has been reported that sponges and dishcloths are both reservoirs and vectors for bacteria in the kitchen [18]. ST693 and ST45 isolates were also isolated from knives and boards before chicken handling, indicating that these bacteria have the potential to spread and survive in the kitchen and might be another important source of cross-contamination. *Campylobacter* has been thought to be vulnerable to extra-intestinal environments; however, Garcia-Sanchez and colleagues demonstrated that after cleaning and disinfection, *Campylobacter* could persist for at least 21 days in a poultry slaughtering plant [52]. The fact that *Campylobacter* was detected on several surfaces after cleaning in our study indicates that it can survive well in kitchen environments. These results highlight the potential for cross-contamination and survival of this pathogen in kitchen environments and the need to raise public awareness on the proper treatment of raw chicken products to avoid foodborne illnesses.

It has been reported that approximately 80% of bacterial infections are associated with biofilm formation because these polymeric structures protect the bacteria against antibiotics, disinfectants, and stressful environmental conditions [53]. Indeed, some strains of *C. jejuni* were able to survive under adverse conditions due to their ability of to form a monospecies biofilm or to integrate into preexisting biofilms [29]. Bacterial biofilm formation in foodservice facilities is a continuous cross-contamination risk because of the survival and persistence of organisms despite disinfectant treatments [54]. Alternatively, the kitchen environment may be consistent with the slaughterhouse environment, in which it was shown that specific clones were selected from a larger population [52]. Notably, three ST693 strains and one ST45 strain isolated from surfaces showed strong biofilm formation ability. Isolates belonging to these two STs caused cross-contamination in the kitchen environment. ST693 strains showed better biofilm formation ability than ST45 strains, which may explain why ST693 was more widely spread in the kitchen environment. Marouani-Gadri and coworkers reported that biofilms play an important role in the contamination of food products in food processing environments [55]. Similarly, biofilm formation ability might be another critical factor affecting the transmission of *Campylobacter* in kitchen environments, and this may be worth considering in the control of *Campylobacter* contamination in commercial kitchens in the future.

## 5. Conclusions

This study surveyed *Campylobacter* cross-contamination between chicken carcasses and sample sites from commercial kitchens and characterized *Campylobacter* isolates by MLST and biofilm formation. Our results revealed that *Campylobacter* is easily transmitted from chicken onto surfaces, where it persists. Sanitary conditions and the biofilm-forming ability of the strains were considered as critical points contributing to the spread of *Campylobacter* in commercial kitchens. Further studies are needed to analyze the risk factors for *Campylobacter* contamination during chicken handling in commercial kitchens, and the potential risk of *Campylobacter* transmitting to cooked foods. New and effective control measures and processing standards are needed to improve handling practices and reduce the risks to human health.

## Figures and Tables

**Table 1:** Samples taken from each of the sampling sites at commercial kitchens in China.

Kitchen	Source of Sample	No. of Samples	Positive Samples	Prevalence (%)
A	Chicken	153	120	78.43
	Sample sites	174	78	44.82
B	Chicken	96	79	82.29
	Sample sites	121	36	29.75
C	Chicken	66	52	78.79
	Sample sites	136	53	38.97
D	Chicken	10	5	50
	Sample sites	17	6	35.29
E	Chicken	38	25	65.79
	Sample sites	31	6	19.35
Total	Chicken	363	281	77.41
	Sample sites	479	179	37.37

**Table 2:** Detection of *Campylobacter* spp. on chicken and sampling sites at commercial kitchens facilities in China.

Sample Source	Procedure	Detection of *Campylobacter* in Commercial Kitchen		
		No. of Positive Samples	Total	Prevalence %
Chicken	Defeathering	41	70	58.57 ^a^
	Evisceration	65	74	87.84 ^b^
	Washing	175	219	79.91 ^b^
	Total	281	363	77.41
Countertop (NFCS*)	Before	2	29	6.90 ^a^
Knife (FCS**)	Before	1	26	3.85 ^a^
Board (FCS)	Before	5	28	17.86 ^a^
Hand (FCS)	Before	7	26	26.92 ^a^
Container (FCS)	Before	6	26	23.08 ^a^
Sink (NFCS)	Before	4	25	16.00 ^a^
Floor (NFCS)	Before	2	26	7.69 ^a^
Total	Before	27	186	14.52
Countertop (NFCS)	After	10	31	32.26 ^a^
Knife (FCS)	After	20	35	57.14 ^ab^
Board (FCS)	After	27	35	77.14 ^b^
Hand (FCS)	After	24	35	68.57 ^b^
Container (FCS)	After	29	35	82.86 ^bc^
Sink (NFCS)	After	4	27	14.81 ^a^
Floor (NFCS)	After	10	30	33.33 ^a^
Total	After	124	228	54.39
Knife (FCS)	Cleaning	10	22	45.45 ^a^
Board (FCS)	Cleaning	12	23	52.17 ^a^
Hand (FCS)	Cleaning	6	20	30.00 ^a^
Total	Cleaning	28	65	43.08

NFCS*: non-food contact surface; FCS**: food contact surface. Different letters indicate significant differences among the groups (*p* < 0.05).

**Table 3:** Counts of *Campylobacter* spp. and total bacterial counts on chicken carcasses and sampling sites in commercial kitchens in China.

Sample Source	Procedure	Loads of *Campylobacter* spp. and Total Bacterial Count (Mean ± SEM log CFU/100 cm^2^)	
		*Campylobacter*	T.B.C.
Chicken carcasses	Defeathering	2.67 ± 0.90 ^a^	6.91 ± 1.20 ^a^
	Evisceration	2.57 ± 0.89 ^a^	6.35 ± 1.39 ^b^
	Washing	2.16 ± 0.80 ^b^	5.89 ± 0.66 ^c^
	Total	2.47 ± 0.86	6.38 ± 1.08
Countertop (NFCS)	Before	1.95 ± 0.35 ^a^	6.29 ± 0.87 ^a^
Knife (FCS)	Before	1.80 ± 0.20 ^a^	6.40 ± 0.87 ^b^
Board (FCS)	Before	2.14 ± 0.24 ^a^	7.03 ± 1.10 ^a^
Hand (FCS)	Before	2.17 ± 0.29 ^a^	6.20 ± 1.04 ^b^
Container (FCS)	Before	2.17 ± 0.31 ^a^	6.36 ± 1.15 ^b^
Sink (NFCS)	Before	2.02 ± 0.16 ^a^	6.37 ± 0.92 ^b^
Floor (NFCS)	Before	1.60 ± 0.00 ^a^	6.07 ± 1.13 ^b^
Total	Before	1.98 ± 0.22	6.29 ± 1.01
Countertop (NFCS)	After	2.01 ± 0.21 ^c^	6.67 ± 0.72 ^bc^
Knife (FCS)	After	2.54 ± 0.23 ^ab^	6.61 ± 0.87 ^bc^
Board (FCS)	After	2.86 ± 0.18 ^a^	7.53 ± 0.74 ^a^
Hand (FCS)	After	2.47 ± 0.19 ^ab^	6.71 ± 0.73 ^bc^
Container (FCS)	After	2.35 ± 0.14 ^b^	6.97 ± 0.86 ^b^
Sink (NFCS)	After	3.04 ± 0.56 ^ab^	6.66 ± 0.95 ^bc^
Floor (NFCS)	After	1.86 ± 0.21 ^c^	6.27 ± 0.94 ^c^
Total	After	2.45 ± 0.25	6.77 ± 0.83
Knife (FCS)	Cleaning	2.33 ± 0.17 ^a^	6.35 ± 0.72 ^b^
Board (FCS)	Cleaning	2.10 ± 0.17 ^a^	7.04 ± 0.83 ^a^
Hand (FCS)	Cleaning	2.44 ± 0.17 ^a^	6.02 ± 0.99 ^ab^
Total	Cleaning	2.29 ± 0.16	6.47 ± 0.85

T.B.C.: total bacterial count. Different letters indicate significant differences among the groups (*p* < 0.05).

## Data Availability

The datasets for this manuscript are not publicly available because the results of this manuscript have not been published yet. Requests to access the datasets should be sent to Honggang Lai, lhghstc@163.com.

## Supplementary Materials

The following are available online at https://www.mdpi.com/article/10.3390/microorganisms9061164/s1, Table S1: The timing of visits to commercial kitchens for sample collection; Table S2: Primer pairs for the identification of *Campylobacter* genus, *Campylobacter jejuni* and *Campylobacter coli*; Table S3: Distribution (%) of *Campylobacter* STs in the procedure of handling chicken in kitchen A.
