# Supplementary material for: An Investigation into the Critical Factors Influencing the Spread of Campylobacter during Chicken Handling in Commercial Kitchens in China
Source: Microorganisms. 2021 May 28;9(6):1164. doi: 10.3390/microorganisms9061164 (PMC8227167; doi:10.3390/microorganisms9061164)
Supplement: Supplementary file 1 [file microorganisms-09-01164-s001.zip › microorganisms-1213410-supplementary.pdf]

microorganisms

MDPI

Supplementary Materials

Table S1. The timing of visits to commercial kitchens for sample collection.

Kitchen	Sample visit in different months					
	January to February	March to April	May to June	July to August	September to October	November to December
A	1	4	1	1	2	4
B	-	4	1	1	1	1
C	-	4	1	1	2	1
D	-	-	1	-	-	-
E	-	-	1	1	-	-

- No visit.

Table S2. Primer pairs for the identification of *Campylobacter* genus, *Campylobacter jejuni* and *Campylobacter coli*.

Primer Name	Oligonucleotide Sequence (5'-3')	Gene	Gene Function	Amplicon Size (bp)
*16SrRNA*1	ATCTAATGGCTTAACCATTAAAC	*16S rRNA*	*Campylobacter* genus	857
*16SrRNA*2	GGACGGTAACTAGTTTAGTATT			
*map*A1	CTATTTTATTTTTGAGTGCTTGTG	*mapA*	*Campylobacter jejuni*	589
*map*A2	GCTTTATTTGCCATTTGTTTTATTA			
*ceu*E1	AATTGAAAAATTGCTCCAACTA	*ceuE*	*Campylobacter coli*	462
*ceu*E2	TGATTTTATTATTTGTAGCAGCG			

Table S3. Distribution (%) of *Campylobacter* STs in the proceed of chicken in kitchen A.

ST* (number/percentage)	Chicken			Before Cutting		After Cutting				Clean Procedure
	Defeather	Eviscerate	Wash	Knife	Board	Knife	Floor	Countertop	Clothes	Knife
ST693	-	-	1 (5.88%)	1 (25%)	2 (66.67%)	-	7 (77.78%)	5 (62.5%)	1 (50%)	1 (50%)
ST45	-	-	5 (29.41%)	-	1 (33.33%)	-	1 (11.11%)	3 (37.5%)	1 (50%)	-
ST8881	2 (40%)	3 (60%)	3 (17.65%)	-	-	-	-	-	-	-
ST8089	-	2 (40%%)	2 (11.76%)	-	-	-	-	-	-	-
ST305	-	-	3 (17.65%)	-	-	-	-	-	-	-
ST10633	-	-	-	2 (50%)	-	-	-	-	-	-
ST4258	1 (20%)	-	-	-	-	-	-	-	-	-
ST7512	1 (20%)	-	-	-	-	-	-	-	-	-
ST10629	1 (20%)	-	-	-	-	-	-	-	-	-
ST137	-	-	1 (5.88%)	-	-	-	-	-	-	-
ST538	-	-	1 (5.88%)	-	-	-	-	-	-	-
ST10318	-	-	1 (5.88%)	-	-	-	-	-	-	-
ST10634	-	-	-	1 (25%)	-	-	-	-	-	-
ST7433	-	-	-	-	-	1 (50%)	-	-	-	-
ST3578	-	-	-	-	-	-	1 (11.11%)	-	-	1 (50%)
ST7433	-	-	-	-	-	1 (50%)	-	-	-	-
Total	5 (100%)	5 (100%)	17 (100%)	4 (100%)	3 (100%)	2 (100%)	9 (100%)	8 (100%)	2 (100%)	2 (100%)

*ST= sequence types; - represented no detected.
